# The long term participation trend for the colorectal cancer screening after the 2011 triple disaster in Minamisoma City, Fukushima, Japan

**DOI:** 10.1038/s41598-021-03225-8

**Published:** 2021-12-13

**Authors:** Hiroaki Saito, Akihiko Ozaki, Michio Murakami, Yoshitaka Nishikawa, Toyoaki Sawano, Sho Fujioka, Yuki Shimada, Tianchen Zhao, Tomoyoshi Oikawa, Yukio Kanazawa, Masaharu Tsubokura

**Affiliations:** 1 Department of Radiation Health Management, Fukushima Medical University School of Medicine, Fukushima, Fukushima Japan; 2 Department of Gastroenterology, Sendai Kousei Hospital, Sendai, Miyagi Japan; 3 Department of Breast Surgery, Jyoban Hospital of Tokiwa Foundation, Iwaki, Fukushima Japan; 4 Research Center for Community Health, Minamisoma Municipal General Hospital, Minamisoma, Fukushima Japan; 5 Department of Health Risk Communication, Fukushima Medical University School of Medicine, Fukushima, Fukushima Japan; 6 Department of Internal Medicine, Soma Central Hospital, Soma, Fukushima Japan; 7 Department of Surgery, Jyoban Hospital of Tokiwa Foundation, Iwaki, Fukushima Japan; 8 Department of Internal Medicine, Soma Central Hospital, Soma, Fukushima Japan; 9 Department of Neurosurgery, Minamisoma Municipal General Hospital, Minamisoma, Fukushima Japan; 10 Department of Gastroenterology, Minamisoma Municipal General Hospital, Minamisoma, Fukushima Japan; 11 Present Address: Center for Infectious Disease Education and Research (CiDER), Osaka University, Suita, Osaka Japan

**Keywords:** Cancer screening, Colorectal cancer, Cancer, Gastroenterology

## Abstract

Colorectal cancer (CRC) screening is a well-established cancer screening method, and its effectiveness depends on maintaining a high participation rate in the target population. In this study, we analyzed the trends in CRC screening participation rates over 10 years in Minamisoma City, where residents were forced to evacuate after the 2011 triple disaster in Fukushima, Japan. The immunochemical fecal occult blood test is provided as municipal CRC screening. We calculated the annual CRC screening participation rate and analyzed the factors associated with participation in screening. Overall, 4069 (12.3%) and 3839 (11.7%) persons participated in CRC screening in 2009 and 2010, respectively; however, the number decreased significantly to 1090 (3.4%) in 2011 when the earthquake occurred. Over the following 3 years, the rate gradually recovered. Multivariable logistic analysis showed that age < 65 years, living alone, and evacuation were significant associated factors for non-participation after 2011 (p < 0.05). In conclusion, the CRC screening participation rate decreased significantly during the Great East Japan Earthquake but recovered over the next 3 years. Further analysis of factors preventing CRC screening participation and research on the long-term effects of its post-disaster decline are important to consider in assessing the need for intervention in post-disaster cancer screening.

## Introduction

Cancer screening aims to promote early cancer detection in an apparently healthy population and to encourage its treatment. Cancer screening effectiveness depends on target population risk, cure possibility upon early cancer detection, and its preventive effect. Colorectal cancer (CRC) is one of those cancers which screening attempts can reduce its numbers and mortality. Therefore, organized CRC screening is provided to residents in many countries^1^. The key indicators for CRC screening quality are the participation rate of residents, colonoscopy compliance rate, the detection rate of polyps, and cancer on colonoscopy^2^. Among them, the CRC screening participation rate varies significantly among countries^1^. Maintaining a high participation rate in CRC screening is an important requirement of local governments that operate screening programs. For effective CRC screening, the European guidelines recommend a participation rate of ≥ 45% of the target population^3^, the American Cancer Society recommends a target of 80%^4,5^, and the Basic Plan to Promote Cancer Control Programs in Japan announced the goal to more than 50%^6^. Elucidating the factors that influence participation rates in CRC screening is vital in achieving well-structured colorectal cancer screening.

Recently, the challenges of cancer care after a natural disaster have received much attention^7–9^, and reducing the impact on efforts to detect cancer and its treatment is important^8^. Natural disasters sometimes result in the disruption of care continuity among previously-diagnosed cancer patients. Hurricane Katrina, which struck New Orleans in 2005, affected more than 1.5 million residents, forcing approximately one-third to evacuate or relocate, including many of the 23,549 cancer patients in the affected areas^10^. Natural disasters can also contribute to a delayed or reduced diagnosis of cancer and therefore to worsened mortality. The number of newly diagnosed cancer patients decreased in the 2 years following Hurricane Katrina^11^, and it is estimated that Hurricane Katrina worsened the subsequent cancer mortality in the affected population^7,10^. This worsened post-disaster cancer mortality has often been reported^8^, possibly due to both the disruption of medical services for previously-diagnosed cancer patients and hampered early cancer diagnosis efforts^9^. Concerns about the impact on cancer care also apply to infectious disease pandemics. The coronavirus disease (COVID-19), which triggered a global pandemic beginning in late 2019, has seemed to make it difficult to provide cancer care to patients in areas with widespread infection^12,13^. The COVID-19 pandemic may have delayed the diagnosis of cancer patients who would otherwise have been diagnosed early and treated^14^. Challenges to cancer treatment and prevention efforts in such disruptive settings are thus highlighted. Meanwhile, there has been insufficient evaluation of the impact of cancer screening, whose effectiveness can take several years or more to verify.

The 9.0 magnitude earthquake and subsequent tsunami that struck eastern Japan on March 11, 2011, followed by the Fukushima nuclear power plant accident, have raised concerns about the long-term health effects of the residents. Thus far, these triple disasters have claimed 19,729 lives and resulted in over 470,000 evacuations in Japan^15^. While most residents could return to their residential areas; some have been forced to evacuate for over a decade. The lifetime cancer risk from nuclear radiation itself is estimated to be very low^16^; however, long-term evacuation has affected the health of the population, increasing the incidence of diabetes, dyslipidemia, and psychological distress^17–19^. Similarly, the impact on medical care has been reported for cancer-related diseases; symptomatic cancer patients delay in seeking medical attention^20^.

In this study, we present the trends in CRC screening participation rate over 10 years before and after the disaster in Minamisoma City, located 14 to 38 km from the nuclear power plant and one of the areas most affected by the disaster. After the disaster, there was a mixture of areas that did or did not require evacuation. Therefore, the population of Minamisoma City consists of groups that were affected in different ways by the evacuation. Surveying the health status of the residents in this area will help to estimate the post-disaster impact of the evacuation on the population. Clarifying the long-term trend in CRC screening in Minamisoma City will enable us to identify the challenges in the medium and long terms after the disaster.

## Methods

### Participants

We conducted a retrospective observational study to analyze the trends in CRC screening participation rate among the citizens listed in the basic resident register of Minamisoma City, Fukushima Prefecture, from 2009 to 2018.

### Locations

Minamisoma City, Fukushima Prefecture, consists of three areas: Odaka, Haramachi, and Kashima Wards (in order of proximity to the Fukushima Daiichi Nuclear Power Plant). On March 12, 2011, the day after the disaster, the government ordered a mandatory evacuation in areas within 20 km of the nuclear power plant. The mandatory evacuation zone included most of Odaka and part of the Haramachi Ward. On March 15, 2011, the government asked residents within 20–30 km from the nuclear power plant to evacuate indoors, and on the 25th, the government ordered a voluntary evacuation. Areas subjected to voluntary evacuation included most of Haramachi and parts of the Odaka and Kashima Wards. On April 22, areas within the 20 km zone in which annual radiation levels of ≥ 20 mSv were expected were declared no-go zones, including parts of Odaka and Haramachi Wards. In April 2012, the no-go zone was divided into three zones: a difficult-to-return zone (no entry), where an annual radiation dose of ≥ 50 mSv was expected; a no-residence zone (temporary return was allowed, but no overnight stay), where an annual radiation dose of 20–50 mSv was expected; and a zone being prepared for lifting of the evacuation order (temporary return was allowed, but not overnight stay; hospitals, welfare facilities, and some stores would be reopened), with an expected annual dose of < 20 mSv. Five years after the disaster, the no-residence zone and the zone prepared for lifting the evacuation order in Minamisoma City were open in July 2016. Minamisoma City’s population, approximately 72,000 in March 2011 before the disaster, was reduced to 11% of that number 11 days after the disaster due to evacuation^21^. After the disaster, hospitals in Minamisoma City, continued to operate except for those in the no-go zone (areas within the 20 km zone in which annual radiation levels of ≥ 20 mSv were expected)^22^; all hospitals and clinics in Odaka Ward were closed just after the disaster, and they have been gradually reopened since 2014^23^.

### CRC screening invitation, method

The main outcome was participation in municipal CRC screening, which was not a mandatory screening, but a screening that the city provided for those who wanted to participate. The Minamisoma city invites residents to participate in CRC screening annually, using the immunochemical fecal occult blood test (iFOBT) by latex agglutination immunoturbidimetry (2 samples). The participants were required to pay 400 yen per person (approximately 3.7 USD). Eligibility for the screening is limited to residents who will be ≥ 40 years old at the end of the fiscal year. In mid-January of each year, eligible residents are mailed a screening application form asking if they wish to participate in CRC screening. These will be sent out at the same time as invitations for other cancers and health examinations. Those who wish to participate in the screening returned the form by mail. At the end of May of the same year, the city sends test containers to those who answer to participate. In the year of the disaster, as in previous years, the invitation to apply was sent out in mid-January before the earthquake, which allowed the recruitment to be done as usual.

From the year following the disaster, the city sent an application form to evacuees outside the city asking if they wished to participate in the CRC screening based on their evacuation registration information. If they wish to participate, they are allowed to participate in the CRC screening at a designated medical institution, at the evacuation destination municipality, or individually at a medical institution, and the Minamisoma city collected the results.

### Data collection

We integrated and analyzed the basic resident register and the CRC screening database. The basic resident register is updated every March and contains IDs that identify individuals, sex, residential ward, age, number of people in the household, and type of evacuation since 2011. The evacuation statuses were categorized as evacuation outside the city, evacuation within the city, and home (not evacuated). Based on the number of household members, a person was assessed as living alone or living with family members (≥ 2 people).

### Analysis

This study was conducted with two targets for each analysis.

#### Analysis of people aged 40–74 years per year

We set the upper age limit of the population to be analyzed to 74 years, according to the recommended age for CRC screening in the United States guidelines^24^. In Japan, there is no upper age limit for the age at which people are recommended to undergo colorectal cancer screening. In order to avoid including very elderly people in the analysis who cannot practically receive screening and to make the results more generalizable, we set an upper age limit for the analysis.

The time-series change in the number of CRC screening participants from 2009 to 2018 was determined for the target population annually. In addition, multivariable logistic analysis was performed on the association of age (40–64, 65–74 years), sex, residential district, and living alone or living with family and participation in CRC screening from 2009 to 2018. Analyses were performed for each year and all years combined. When all years were combined, random-effects analysis was used. In addition, we examined the association between evacuation status and participation in CRC screening in addition to the aforementioned factors, focusing on the years 2011–2018. Evacuation status was analyzed in terms of the following three conditions: (a) not evacuated vs. evacuated, (b) not evacuated vs. evacuated to the city vs. evacuated to outside the city, and (c) not evacuated plus evacuated to the city vs. evacuated to outside the city. We performed multivariable logistic regression analysis for each year and all years combined and stratified the analysis by evacuation status.

#### Analysis by groups according to screening adherence

We include the residents for whom enrollment data were available for all years from 2009 to 2018 and who were 64 years old or younger in 2009 (74 years old or younger in 2018) in the analysis. Based on screening participation in 2009 and 2010, the residents were divided into three groups: those who participated in (1) both 2009 and 2010, (2) either 2009 or 2010, (3) neither 2009 nor 2010. We analyzed the difference in orientation toward CRC screening among individuals and the change in participation in CRC screening after the disaster.

The analysis was performed using STATA ver 15.0 (Stata Corp), and a p-value < 0.05 was considered statistically significant. The study protocol was approved by the ethics committees of the Minamisoma Municipal General Hospital and Fukushima Medical University (approval nos. 2-20 and 3065, respectively). Because this is a retrospective study, the opt-out approach was used to obtain informed consent. All methods were performed in accordance with the Declarations of Helsinki.

## Results

Overall, 44,766 residents were included in the 10-year analysis from 2009 to 2018. Of these, 21,164 (47.3%) were female, and the mean age in 2009 was 52.7 ± 12.0 years. The characteristics of the residents for each year are shown in Supplementary Table S1 and Supplementary Figs. S1 and S2.

### Annual participation rates of CRC screening

**Figure 1:**
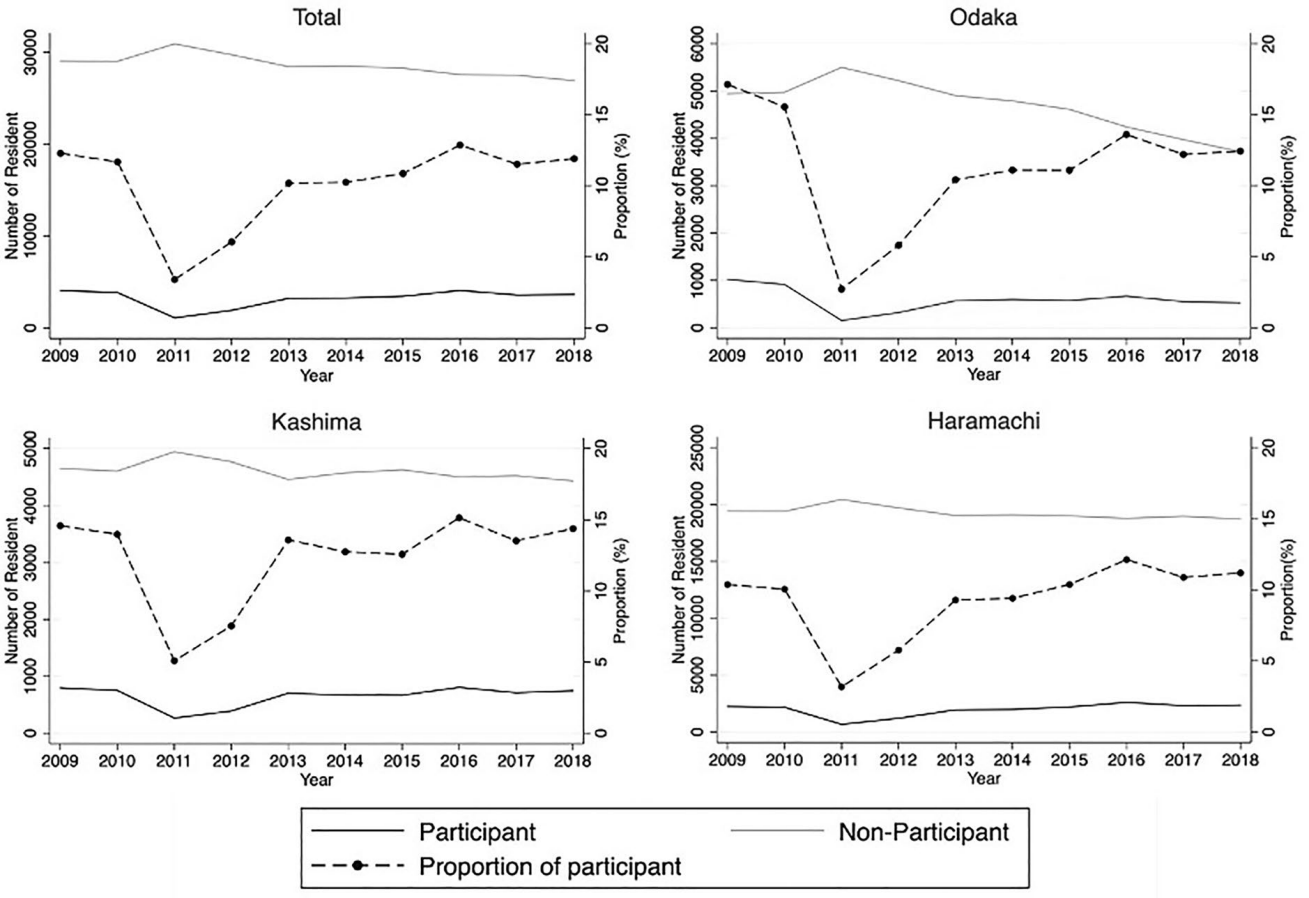
Trends in participation in CRC screening in Minamisoma City. The dashed lines represent participation rates. The thin line represents the number of people who did not participate, and the thick line represents the number of people who did participate.

Of the target population, 4069 (12.3%) and 3839 (11.7%) residents participated in 2009 and 2010, respectively; however, the number decreased significantly to 1090 (3.4%) in 2011 when the disaster occurred (Fig. 1). In 2010, 2172 (10.0%), 750 (14.0%), and 917 (15.6%) residents underwent CRC screening in Haramachi, Kashima, and Odaka Wards, respectively, and these numbers decreased to 671 (3.2%), 265 (5.1%), and 154 (2.7%), respectively, in 2011 (Supplementary Table S2).

### Impact of factors on CRC screening participation rate

Multivariable regression analysis showed that male sex, age < 65 years, and living alone were significantly associated with non-participation; residents in Haramachi Ward were less likely to participate compared to those in Kashima Ward; and residents in Odaka Ward were likelier to participate in both 2009 and 2010 (Table 1, Supplementary Table S3). Factors other than residential district were similar after 2011; however, residence in Haramachi and Odaka Wards were both factors associated with non-participation, compared to the residence in Kashima.

**Table 1:** Multivariable logistic regression analysis for the CRC screening participation.

	2009		2010		2011		2012		2013		2014		2015		2016		2017		2018	
	Odds (95% CI)	p	Odds (95% CI)	p	Odds (95% CI)	p	Odds (95% CI)	p	Odds (95% CI)	p	Odds (95% CI)	p	Odds (95% CI)	p	Odds (95% CI)	p	Odds (95% CI)	p	Odds (95% CI)	p
**Sex (Reference = Female)**																				
Male	0.68 (0.64–0.73)	< 0.001	0.65 (0.60–0.69)	< 0.001	0.66 (0.58–0.75)	< 0.001	0.70 (0.64–0.77)	< 0.001	0.64 (0.60–0.69)	< 0.001	0.66 (0.61–0.71)	< 0.001	0.62 (0.58–0.67)	< 0.001	0.55 (0.51–0.58)	< 0.001	0.58 (0.54–0.63)	< 0.001	0.54 (0.51–0.58)	< 0.001
**Age (Reference = 65–74 yr)**																				
40- 64 yr	0.34 (0.32–0.36)	< 0.001	0.35 (0.32–0.37)	< 0.001	0.37 (0.32–0.41)	< 0.001	0.29 (0.27–0.32)	< 0.001	0.34 (0.31–0.36)	< 0.001	0.32 (0.30–0.34)	< 0.001	0.32 (0.30–0.35)	< 0.001	0.34 (0.32–0.36)	< 0.001	0.29 (0.27–0.31)	< 0.001	0.29 (0.27–0.31)	< 0.001
**Residence (Reference = Kashima)**																				
Haramachi	0.68 (0.62–0.74)	< 0.001	0.68 (0.62–0.75)	< 0.001	0.61 (0.53–0.71)	< 0.001	0.75 (0.67–0.85)	< 0.001	0.64 (0.58–0.71)	< 0.001	0.71 (0.64–0.78)	< 0.001	0.8 (0.73–0.88)	< 0.001	0.78 (0.71–0.85)	< 0.001	0.79 (0.72–0.86)	< 0.001	0.76 (0.69–0.83)	< 0.001
Odaka	1.21 (1.09–1.34)	< 0.001	1.14 (1.02–1.26)	0.019	0.52 (0.42–0.64)	< 0.001	0.77 (0.66–0.89)	0.001	0.74 (0.66–0.83)	< 0.001	0.86 (0.76–0.97)	0.011	0.85 (0.75–0.96)	0.009	0.87 (0.77–0.97)	0.014	0.88 (0.78–1.00)	0.041	0.83 (0.73–0.94)	0.003
**Household (Reference = Multiple)**																				
Alone	0.58 (0.50–0.67)	< 0.001	0.59 (0.51–0.69)	< 0.001	0.61 (0.47–0.78)	< 0.001	0.63 (0.52–0.75)	< 0.001	0.67 (0.59–0.78)	< 0.001	0.63 (0.55–0.73)	< 0.001	0.58 (0.5–0.66)	< 0.001	0.55 (0.49–0.63)	< 0.001	0.59 (0.52–0.67)	< 0.001	0.62 (0.55–0.70)	< 0.001

*Odds* odds ratio, *yr* years old.

In the analysis with evacuation as a factor, CRC screening non-participation was associated with evacuation in all years from 2011 to 2018 (Supplementary Table S4). Furthermore, by type of evacuation, both in-city and out-of-city evacuation were associated with screening non-participation compared to non-evacuation, and out-of-city evacuation was associated with screening  non-participation compared to in-city evacuation or non-evacuation (Supplementary Table S5).

In the stratified analysis by type of evacuation, male sex, age 40–64 years, living in Haramachi Ward, and living alone were associated with CRC screening non-participation when the residents were divided into living home, in-city evacuees, and out-of-city evacuees, as well as when they were divided into living home and evacuees, and living in the city and living outside of the city (Table 2).

**Table 2:** Stratified analysis of CRC screening participation between different evacuation status.

	Non-evacuation vs inside of the city vs Outside of the city*						Non-evacuation vs evacuation				Inside of the city vs outside of the city**			
	Non-evacuation		Inside of the city		Outside of the city		Non-evacuation		Evacuation		Inside of the city		Outside of the city	
	Odds (95% CI)	p	Odds (95% CI)	p	Odds (95% CI)	p	Odds (95% CI)	p	Odds (95% CI)	p	Odds (95% CI)	p	Odds (95% CI)	p
**Year (Reference = 2018)**														
2011	0.08 (0.07–0.10)	< 0.001	0.06 (0.04–0.09)	< 0.001	0.18 (0.14–0.23)	< 0.001	0.08 (0.07–0.10)	< 0.001	0.08 (0.07–0.10)	< 0.001	0.08 (0.07–0.09)	< 0.001	0.18 (0.14–0.23)	< 0.001
2012	0.20 (0.18–0.23)	< 0.001	0.18 (0.15–0.23)	< 0.001	0.37 (0.29–0.47)	< 0.001	0.20 (0.18–0.23)	< 0.001	0.21 (0.18–0.25)	< 0.001	0.20 (0.18–0.22)	< 0.001	0.36 (0.28–0.46)	< 0.001
2013	0.61 (0.56–0.68)	< 0.001	0.63 (0.53–0.76)	< 0.001	0.85 (0.67–1.07)	0.169	0.61 (0.56–0.68)	< 0.001	0.64 (0.56–0.73)	< 0.001	0.62 (0.57–0.68)	< 0.001	0.85 (0.67–1.07)	0.170
2014	0.59 (0.54–0.65)	< 0.001	0.65 (0.55–0.78)	< 0.001	0.97 (0.77–1.23)	0.820	0.59 (0.54–0.65)	< 0.001	0.70 (0.61–0.80)	< 0.001	0.61 (0.56–0.66)	< 0.001	0.97 (0.77–1.23)	0.803
2015	0.75 (0.69–0.83)	< 0.001	0.64 (0.54–0.77)	< 0.001	1.00 (0.79–1.27)	0.978	0.75 (0.69–0.83)	< 0.001	0.72 (0.63–0.83)	< 0.001	0.73 (0.68–0.80)	< 0.001	1.01 (0.80–1.27)	0.952
2016	1.15 (1.06–1.26)	0.002	1.19 (1.01–1.40)	0.036	1.56 (1.24–1.96)	< 0.001	1.15 (1.06–1.26)	0.002	1.22 (1.07–1.39)	0.002	1.17 (1.08–1.26)	< 0.001	1.56 (1.24–1.97)	< 0.001
2017	0.86 (0.78–0.94)	0.001	0.89 (0.75–1.04)	0.138	1.17 (0.92–1.49)	0.204	0.86 (0.78–0.94)	0.001	0.95 (0.83–1.08)	0.425	0.87 (0.80–0.94)	< 0.001	1.17 (0.92–1.49)	0.206
**Sex (Reference = Female)**														
Male	0.31 (0.28–0.35)	< 0.001	0.33 (0.27–0.40)	< 0.001	0.52 (0.43–0.62)	< 0.001	0.31 (0.28–0.35)	< 0.001	0.39 (0.34–0.46)	< 0.001	0.32 (0.29–0.35)	< 0.001	0.49 (0.41–0.59)	< 0.001
**Age (Reference = 65–74 yr)**														
40–64 yr	0.25 (0.23–0.27)	< 0.001	0.26 (0.22–0.30)	< 0.001	0.33 (0.28–0.39)	< 0.001	0.25 (0.23–0.27)	< 0.001	0.30 (0.27–0.34)	< 0.001	0.25 (0.23–0.27)	< 0.001	0.33 (0.28–0.39)	< 0.001
**Residence (Reference = Kashima)**														
Haramachi	0.61 (0.54–0.70)	< 0.001	0.50 (0.38–0.66)	< 0.001	0.58 (0.40–0.85)	0.005	0.61 (0.54–0.70)	< 0.001	0.57 (0.45–0.71)	< 0.001	0.60 (0.53–0.68)	< 0.001	0.59 (0.42–0.83)	0.003
Odaka	0.16 (0.02–1.61)	0.119	1.03 (0.78–1.35)	0.858	0.76 (0.52–1.12)	0.169	0.16 (0.02–1.61)	0.119	0.89 (0.71–1.11)	0.294	0.91 (0.77–1.08)	0.293	0.77 (0.54–1.09)	0.138
**Household (Reference = Multiple)**														
Alone	0.45 (0.38–0.53)	< 0.001	0.41 (0.30–0.54)	< 0.001	0.68 (0.50–0.94)	< 0.001	0.45 (0.38–0.53)	< 0.001	0.47 (0.37–0.58)	< 0.001	0.45 (0.39–0.52)	< 0.001	0.55 (0.40–0.76)	< 0.001

*Odds* odds ratio, *yr* years old.

*Stratified by those not under evacuation, those evacuated within the city, and those evacuated outside the city.

**Comparison of those who evacuated outside the city and those who are in the city including those who did not evacuate and those who evacuated to the city.

### Participation trends by adherence to pre-disaster CRC screening

**Figure 2:**
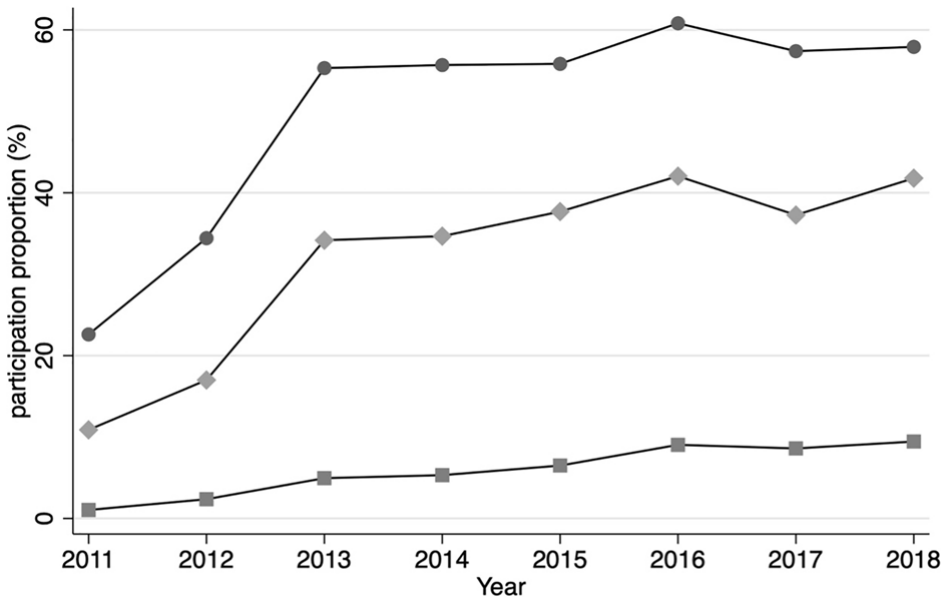
Participation trends by tendency to undergo CRC screening before the disaster. The line graphs with round dots show the proportions of those who participated in CRC screening in both 2009 and 2010 before the earthquake, while the line graphs with diamonds show the proportions of those who participated CRC screening either in 2009 or 2010, and the line graphs with squares show the proportions of those who did not participate CRC screening in 2009 or 2010.

Of 20,835 people who continued to register as residents in Minamisoma from 2009 to 2018, 1345 (6.5%) underwent screening in 2009 and 2010, 1194 (5.7%) in only one year, and 18,296 (87.8%) had never undergone screening. The group that underwent colorectal screening in both two years had the highest uptake rate in 2011 compared with the group that underwent colorectal screening only in one year or never (22.6% vs. 10.9% vs. 1.0%) (Fig. 2). In the entire period analysis, male sex and evacuations were significantly associated with non-participation in all groups; only for the group that never participated, living alone was significant, which appeared from 2015 (Tables 3, 4, 5, Supplementary Table S6).

**Table 3:** Multivariable logistic regression analysis for the CRC screening participation among those who participated CRC screening in both 2009 and 2010.

Variable	2011		2012		2013		2014		2015		2016		2017		2018	
	Odds (95% CI)	p	Odds (95% CI)	p	Odds (95% CI)	p	Odds (95% CI)	p	Odds (95% CI)	p	Odds (95% CI)	p	Odds (95% CI)	p	Odds (95% CI)	p
**Age (Reference = 60–64 yr)**																
40–49 yr	0.72 (0.48–1.08)	0.114	0.37 (0.25–0.55)	< 0.001	0.59 (0.43–0.82)	0.002	0.64 (0.46–0.88)	0.007	0.43 (0.31–0.60)	< 0.001	0.65 (0.47–0.91)	0.011	0.53 (0.38–0.73)	< 0.001	0.50 (0.36–0.69)	< 0.001
50–59 yr	0.94 (0.71–1.25)	0.671	0.70 (0.55–0.90)	0.005	0.81 (0.63–1.03)	0.081	0.89 (0.70–1.13)	0.343	0.87 (0.68–1.11)	0.258	0.95 (0.74–1.21)	0.666	0.74 (0.58–0.94)	0.014	1.01 (0.80–1.29)	0.917
**Sex (Reference = Female)**																
Male	0.66 (0.49–0.88)	0.005	0.80 (0.62–1.02)	0.071	0.75 (0.59–0.94)	0.015	0.84 (0.66–1.06)	0.137	0.71 (0.56–0.90)	0.004	0.76 (0.60–0.96)	0.020	0.75 (0.60–0.95)	0.018	0.72 (0.57–0.91)	0.005
**Residence (Reference = Kashima)**																
Haramachi	0.72 (0.53–0.98)	0.038	0.99 (0.74–1.31)	0.921	0.90 (0.68–1.21)	0.492	0.72 (0.54–0.97)	0.028	1.07 (0.80–1.42)	0.651	0.89 (0.66–1.19)	0.431	0.87 (0.66–1.16)	0.355	0.93 (0.70–1.23)	0.616
Odaka	0.56 (0.36–0.88)	0.012	0.81 (0.53–1.24)	0.340	0.74 (0.49–1.11)	0.144	0.64 (0.42–0.96)	0.030	0.88 (0.58–1.35)	0.570	0.60 (0.39–0.92)	0.019	0.60 (0.39–0.92)	0.018	0.72 (0.47–1.10)	0.125
**Household (Reference = Multiple)**																
Alone	1.03 (0.57–1.87)	0.928	0.68 (0.40–1.16)	0.160	1.39 (0.87–2.22)	0.173	1.14 (0.73–1.76)	0.563	0.99 (0.64–1.52)	0.965	0.88 (0.58–1.32)	0.531	0.93 (0.62–1.38)	0.714	1.16 (0.79–1.71)	0.451
**Evacuation status (Reference = non-evacuation)**																
Evacuation	0.49 (0.36–0.66)	< 0.001	0.66 (0.48–0.89)	0.0007	0.47 (0.34–0.63)	< 0.001	0.59 (0.43–0.81)	0.001	0.50 (0.36–0.70)	< 0.001	0.64 (0.46–0.89)	0.008	0.66 (0.47–0.92)	0.014	0.83 (0.60–1.16)	0.278

*Odds* odds ratio, *yr* years old.

**Table 4:** Multivariable logistic regression analysis for the CRC screening participation among those who participated CRC screening either in 2009 or 2010.

Variable	2011		2012		2013		2014		2015		2016		2017	2018		
	Odds (95% CI)	p	Odds (95% CI)	p	Odds (95% CI)	p	Odds (95% CI)	p	Odds (95% CI)	p	Odds (95% CI)	p	Odds (95% CI)	p	Odds (95% CI)	p
**Age (Reference = 60–64 yr)**																
40–49 yr	0.49 (0.27–0.90)	0.021	0.24 (0.14–0.41)	< 0.001	0.45 (0.31–0.64)	< 0.001	0.35 (0.24–0.50)	< 0.001	0.37 (0.26–0.53)	< 0.001	0.34 (0.24–0.48)	< 0.001	0.41 (0.29–0.59)	< 0.001	0.34 (0.24–0.48)	< 0.001
50–59 yr	0.99 (0.67–1.47)	0.950	0.51 (0.37–0.72)	< 0.001	0.79 (0.61–1.03)	0.085	0.76 (0.58–0.99)	0.042	0.82 (0.63–1.06)	0.126	0.79 (0.61–1.02)	0.075	0.82 (0.63–1.07)	0.142	0.67 (0.51–0.87)	0.002
**Sex (Reference = Female)**																
Male	0.73 (0.49–1.09)	0.121	0.82 (0.59–1.13)	0.215	0.99 (0.77–1.27)	0.907	0.95 (0.74–1.22)	0.668	0.90 (0.70–1.15)	0.396	0.76 (0.60–0.97)	0.029	0.75 (0.59–0.96)	0.023	0.71 (0.56–0.91)	0.007
**Residence (Reference = Kashima)**																
Haramachi	0.60 (0.39–0.93)	0.021	0.94 (0.64–1.38)	0.743	0.80 (0.59–1.08)	0.146	0.96 (0.70–1.30)	0.773	0.98 (0.73–1.33)	0.914	1.03 (0.77–1.40)	0.825	0.99 (0.73–1.34)	0.940	0.86 (0.64–1.16)	0.326
Odaka	0.59 (0.31–1.13)	0.110	1.21 (0.65–2.27)	0.547	0.72 (0.44–1.16)	0.176	0.80 (0.49–1.29)	2.359	1.15 (0.71–1.87)	2.572	1.06 (0.66–1.70)	0.824	1.08 (0.66–1.76)	0.761	0.75 (0.46–1.21)	0.237
**Household (Reference = Multiple)**																
Alone	1.66 (0.75–3.67)	0.209	1.45 (0.74–2.86)	0.282	0.91 (0.52–1.58)	0.726	0.88 (0.50–1.53)	0.645	0.75 (0.43–1.31)	0.311	0.83 (0.49–1.40)	0.480	1.07 (0.66–1.74)	0.788	0.92 (0.57–1.48)	0.735
**Evacuation status (Reference = Non-Evacuation)**																
Evacuation	0.75 (0.49–1.14)	0.175	0.51 (0.32–0.79)	0.003	0.63 (0.45–0.89)	0.009	0.81 (0.58–1.15)	0.244	0.60 (0.42–0.87)	0.006	0.72 (0.51–1.02)	0.066	0.72 (0.50–1.03)	0.071	0.68 (0.48–0.97)	0.031

*Odds* odds ratio, *yr* years old.

**Table 5:** Multivariable logistic regression analysis for the CRC screening participation among those who did not participate CRC screening in 2009 or 2010.

Variable	2011		2012		2013		2014		2015		2016		2017		2018	
	Odds (95% CI)	p	Odds (95% CI)	p	Odds (95% CI)	p	Odds (95% CI)	p	Odds (95% CI)	p	Odds (95% CI)	p	Odds (95% CI)	p	Odds (95% CI)	p
**Age (Reference = 60–64 yr)**																
40–49 yr	0.22 (0.14–0.35)	< 0.001	0.23 (0.17–0.30)	< 0.001	0.31 (0.25–0.37)	< 0.001	0.25 (0.20–0.31)	< 0.001	0.26 (0.21–0.31)	< 0.001	0.33 (0.28–0.38)	< 0.001	0.28 (0.24–0.32)	< 0.001	0.28 (0.24–0.33)	< 0.001
50–59 yr	0.53 (0.39–0.72)	< 0.001	0.49 (0.40–0.60)	< 0.001	0.64 (0.55–0.75)	< 0.001	0.67 (0.58–0.77)	< 0.001	0.63 (0.55–0.72)	< 0.001	0.77 (0.68–0.86)	< 0.001	0.73 (0.65–0.82)	< 0.001	0.72 (0.64–0.80)	< 0.001
**Sex (Reference = Female)**																
Male	0.74 (0.56–0.99)	0.043	0.77 (0.64–0.94)	0.008	0.65 (0.57–0.75)	< 0.001	0.74 (0.65–0.85)	< 0.001	0.73 (0.65–0.83)	< 0.001	0.62 (0.56–0.68)	< 0.001	0.70 (0.63–0.77)	< 0.001	0.67 (0.61–0.74)	< 0.001
**Residence (Reference = Kashima)**																
Haramachi	0.68 (0.48–0.98)	0.038	0.79 (0.61–1.01)	0.056	0.72 (0.61–0.86)	< 0.001	0.78 (0.66–0.92)	0.004	0.82 (0.71–0.96)	0.014	0.84 (0.73–0.96)	0.010	0.86 (0.75–0.99)	0.032	0.86 (0.75–0.98)	0.020
Odaka	0.83 (0.50–1.37)	0.457	0.84 (0.58–1.23)	0.378	0.91 (0.70–1.18)	0.466	1.09 (0.84–1.42)	0.505	1.06 (0.82–1.36)	0.679	1.03 (0.83–1.28)	0.771	0.96 (0.77–1.20)	0.717	0.95 (0.76–1.19)	0.659
**Household (Reference = Multiple)**																
Alone	0.90 (0.52–1.55)	0.696	0.75 (0.51–1.10)	0.137	0.86 (0.66–1.10)	0.227	0.80 (0.62–1.02)	0.069	0.79 (0.63–0.98)	0.031	0.75 (0.62–0.90)	0.002	0.71 (0.59–0.86)	< 0.001	0.74 (0.62–0.88)	0.001
**Evacuation status (Reference = Non-Evacuation)**																
Evacuation	1.04 (0.75–1.44)	0.814	0.97 (0.76–1.24)	0.786	0.97 (0.81–1.17)	0.771	0.88 (0.73–1.05)	0.161	0.75 (0.62–0.89)	0.001	0.82 (0.70–0.95)	0.008	0.88 (0.76–1.02)	0.099	0.80 (0.69–0.93)	0.003

*Odds* odds ratio, *yr* years old.

## Discussion

This study aimed to estimate the impact of the Great East Japan Earthquake and subsequent disasters on CRC screening among residents of Minamisoma City, which was particularly affected by the disaster. We analyzed the results of CRC screening for 10 years before and after the disaster and showed that the disaster had significantly impacted the participation rate of CRC screening.

This study was not designed to elucidate the reasons for the decrease in post-earthquake cancer screening uptake. However, in the multivariable regression analysis, evacuation after the disaster was a significant factor for non-participation in CRC screening. Even among those who had been screened for two consecutive years before the disaster, those who had been evacuated in 2011 showed a decrease in screening uptake with an odds ratio of 0.49. The main reason for the decline in CRC screening participation might be that CRC screening, a preventive initiative, became less priority for residents after the disaster. In fact, in the areas affected by the Great East Japan Earthquake, it has been reported that evacuees were less proactive in seeking medical services after the disaster. For example, symptomatic breast cancer patients and CRC patients have been reported to delay in seeking hospital care^20,25–27^. Thus, it is reasonable to assume that the priority of screening people without symptoms has been further reduced.

CRC screening participation rate declined the most in the year of the disaster, gradually improved in the subsequent years. Three years after the disaster, the participation rate almost recovered to the pre-disaster level. This slow recovery in the participation rate symbolizes the magnitude of the impact of this disaster. After the disaster, many people in Minamisoma City were forced to evacuate. Some people were able to move to the homes of acquaintances, while others moved into temporary housing, and the living environment of the residents changed drastically after the disaster^21^. The long-term evacuation and lifestyle change that residents were forced to endure is thought to have had a prolonged impact on CRC screening participation rate in Minamisoma City. The trend in screening participation rates shown in this study seems consistent with the tendency to refrain from usual health maintenance services for chronic diseases and other decreases after a major disaster. For example, in the three years following Hurricane Katrina, the proportion of residents undergoing diabetes care was lower than in the pre-disaster period^28^; mental health utilization among the United States military veterans also declined in the months following the disaster^29^. After a large-scale disaster, both short-term and long-term medical interruptions are important, and recovery efforts need to be focused on. Our study results support this finding.

In this study, living alone was a significant factor associated with less participation both before and after the disaster. Partners, family members, and friends are known to promote participation in CRC screening, and our study results seem to align with previous studies^30,31^. The post-disaster impact of the altered community formed by family and friends on cancer treatment should be noted. For example, the absence of family members and friends, who had previously pointed out changes in symptoms and health status, was cited as a reason for the delay in consultation of symptomatic cancer patients after the disaster^20,25,27^. In this study, it was unclear whether these factors had a strong impact on screening participation, particularly after the disaster; this state of evacuation may further deepen the social isolation of single people. Such disincentives to participate in CRC screening may improve with the promotion of community building in the evacuation sites after the disaster and the approach to participation in screening.

Interestingly, there was a change in the tendency to undergo CRC screening in each district before and after the disaster. Odaka Ward had a unique characteristic because it had the highest CRC screening participation rate among the three districts before the disaster but had a lower participation rate than Kashima Ward after the disaster. Odaka Ward is the closest to the nuclear power plant in Minamisoma City, and most of its residents were forced to evacuate. In addition, most of the evacuation orders in those areas were lifted in 2016, five years after the disaster, and the slowest among the three districts. Medical institutions and other services have gradually resumed after the disaster^23^; however, post-evacuation residents may have lost their ties to medical institutions that they had regularly visited since the early post-earthquake period. Physician recommendations and regular visits to medical institutions positively affect the participation rate of CRC screening^32^, availability to a medical institution capable of performing colonoscopy may have provided a good recommendation for CRC screening, and the failure to reestablish such connections upon return from evacuation indicates that barriers to health maintenance and participation in cancer screenings may not be removed.

In our analysis, although the CRC screening participation rate in Minamisoma City returned to the pre-disaster level after 2013, the overall CRC screening participation rate remained constant at the 10% level. The problem of low participation rates has been addressed, and improvements are being made in many countries. For example, Spain successfully increased the rate from 8.5 to 31.8% between 2009 and 2017^33^. Similarly, in the overall trend in Japan, awareness of participation in CRC screening has increased, and the participation rate has been increasing yearly. According to the questionnaire-based survey, the participation rate of colorectal cancer screening among men and women aged 40 to 69 in Japan in 2010 was reported to be 28.1% and 23.9%, respectively, which increased to 41.4% and 34.5%, respectively, in 2013^34^. The fact that the screening rate in Minamisoma City has only risen to the pre-earthquake standard after the disaster may indicate that the effects of the disaster have not been resolved. In order to increase the participation rate in CRC screening, the solutions such as reducing the cost of iFOBT, the individual burden of colonoscopy in case of a positive iFOBT result, and disseminating public education on colorectal cancer should be actively adopted.

Long-term observation of the changes in CRC incidence and treatment caused by changes in CRC screening participation rate is necessary. A decrease in the participation in CRC screening is expected to prevent a decrease in CRC incidence that could have been prevented. Although the participation rate in CRC screening was originally low in this area and the impact of the decrease in the participation rate due to the disaster may be small, it is necessary to estimate the impact of this change on the health of the residents. It would be important to calculate it using indicators such as life expectancy loss that are general enough to compare with the impact of other environmental changes. This work will also be important in determining the priority of cancer screening projects in the acute, medium, and long-term phases of a disaster on a similar scale.

Our study had several limitations. First, some of the individual factors, such as access to medical institutions and the history of CRC, were not assessed. The evacuation and environmental changes caused by the disaster may have altered access to health care facilities, which in turn may have affected the CRC screening participation rate. Although such information is not available from the current data, it is one of the important factors and should be analyzed in future studies.

Second, the actual status of CRC screening other than municipal CRC screening is unknown. In Japan, two major other types of CRC screening are performed: occupational screening provided to workers and screening that individual receive of their own free will. The presence or absence of participation in other types of CRC screening was not analyzed in our study. However, since non-municipal CRC screening is provided by private companies and individual medical institutions, it is difficult to make a uniform count. In order to clarify these figures, it is necessary to use the questionnaire survey of residents, and it is a challenge to further study. In addition, municipal CRC screening conducted by local governments is the most widely utilized screening with a large target population, and the analysis of municipal CRC screening is meaningful for analyzing the trend in residents’ participation in screening. Third, we could not use data on socioeconomic status, which is one of the factors related to colorectal cancer screening participation, such as annual income, educational level, marital status, work status, and occupation. The identification of socioeconomic status that is susceptible to the effects of disasters is an issue for future research. Finally, because the analysis of this study focused on participation in primary screening, it is not known regarding the positive rate of fecal occult blood or the performance of subsequent colonoscopy. These results must be included in order to accurately estimate the future impact of reduced participation in screening.

In conclusion, the CRC screening participation rate in Minamisoma City, which was greatly affected by the Great East Japan Earthquake, decreased significantly at the year of the disaster but recovered over the next 3 years. Further analysis of factors preventing participation in screening and research on the long-term effects of the post-disaster decline in CRC screening participation are important to consider in assessing the need for intervention in post-disaster cancer screening.

## Supplementary Information

Supplementary Information.

## Data Availability

The data that support the findings of this study are available from the corresponding author, HS, upon reasonable request.
