# Supplementary material for: The long term participation trend for the colorectal cancer screening after the 2011 triple disaster in Minamisoma City, Fukushima, Japan
Source: Sci Rep. 2021 Dec 13;11:23851. doi: 10.1038/s41598-021-03225-8 (PMC8668878; doi:10.1038/s41598-021-03225-8)
Supplement: Supplementary file 1 — Supplementary Information. [file 41598_2021_3225_MOESM1_ESM.docx]

Supplementary Table 1 The characteristics of the residents eligible for the CRC screening in Minamisoma City for each year

| Year | 2009 | 2010 | 2011 | 2012 | 2013 | 2014 | 2015 | 2016 | 2017 | 2018 |
| --- | --- | --- | --- | --- | --- | --- | --- | --- | --- | --- |
| Residents | 33,103 | 32,851 | 31,996 | 31,634 | 31,641 | 31,742 | 31,709 | 31,633 | 31,074 | 30,544 |
| Male, n (%) | 16,487 (49.8) | 16,386 (49.9) | 15,968 (49.9) | 15,851 (50.1) | 15,908 (50.3) | 16,062 (50.6) | 16,188 (51.1) | 16,239 (51.3) | 15,939 (51.3) | 15,707 (51.4) |
| Female, n (%) | 16,616 (50.2) | 16,465 (50.1) | 16,028 (50.1) | 15,783 (49.9) | 15,733 (49.7) | 15,680 (49.4) | 15,521 (49.0) | 15,394 (48.7) | 15,135 (48.7) | 14,837 (48.6) |
| Age, mean (SD) | 57.4 (9.5) | 57.5 (9.5) | 57.6 (9.5) | 57.7 (9.5) | 57.8 (9.7) | 57.9 (9.8) | 57.9 (9.9) | 57.9 (9.9) | 58.1 (10.0) | 58.2 (10.0) |
| Evacuation n (%) | - | - | 16,858 (52.7) | 12,692 (40.1) | 11,966 (37.8) | 11,529 (36.3) | 11,227 (35.4) | 10,892 (34.4) | 10,315 (33.2) | 10,282 (33.7) |
| Household |  |  |  |  |  |  |  |  |  |  |
| Alone, n (%) | 2,886 (8.7) | 2,871 (8.7) | 2,978 (9.3) | 3,094 (9.8) | 3,211 (10.2) | 3,438 (10.8) | 3,753 (11.8) | 4,090 (12.9) | 4,058 (13.1) | 4,102 (13.4) |
| With family, n (%) | 30,217 (91.3) | 29,980 (91.3) | 29,018 (90.7) | 28,540 (90.2) | 28,430 (89.9) | 28,304 (89.2) | 27,956 (88.2) | 27,543 (87.1) | 27,016 (86.9) | 26,442 (86.6) |
| Residence |  |  |  |  |  |  |  |  |  |  |
| Kashima, n (%) | 5,454(16.5) | 5,361(16.3) | 5,209(16.3) | 5,160(16.3) | 5,165(16.3) | 5,250(16.5) | 5,301(16.7) | 5,312(16.8) | 5,239(16.9) | 5,184(17.0) |
| Haramachi, n (%) | 21,680(65.5) | 21,596(65.7) | 21,131(66.0) | 20,930(66.2) | 20,998(66.4) | 21,100(66.5) | 21,217(66.9) | 21,407(67.7) | 21,306(68.6) | 21,108(69.1) |
| Odaka, n (%) | 5,969(18.0) | 5,894(17.9) | 5,656(17.7) | 5,544(17.5) | 5,478(17.3) | 5,392(17.0) | 5,191(16.4) | 4,914(15.5) | 4,529(14.6) | 4,252(13.9) |

Supplementary Table 2 Number of participants and the participation rate by the category

| Variables |  | 2009 | 2010 | 2011 | 2012 | 2013 | 2014 | 2015 | 2016 | 2017 | 2018 |
| --- | --- | --- | --- | --- | --- | --- | --- | --- | --- | --- | --- |
| Sex |  |  |  |  |  |  |  |  |  |  |  |
| Male |  | 1,667 (10.1) | 1532 (9.3) | 432 (2.7) | 795 (5.0) | 1305 (8.2) | 1336 (8.3) | 1,379 (8.5) | 1,540 (9.5) | 1401 (8.8) | 1,379 (8.8) |
| Female |  | 2,402 (14.5) | 2307 (14.0) | 658 (4.1) | 1121 (7.1) | 1919 (12.2) | 1919 (12.2) | 2,067 (13.3) | 2,532 (16.4) | 2179 (14.4) | 2,260 (15.2) |
| Age |  |  |  |  |  |  |  |  |  |  |  |
| 40-64yr |  | 2,166 (8.9) | 2089 (8.5) | 576 (2.4) | 889 (3.9) | 1584 (7.0) | 1487 (6.7) | 1,549 (7.1) | 1,849 (8.6) | 1448 (7.0) | 1,460 (7.3) |
| 65-74yr |  | 1,903 (22) | 1750 (20.9) | 514 (6.3) | 1027 (11.9) | 1640 (18) | 1768 (18.3) | 1,897 (19.1) | 2,223 (21.7) | 2132 (20.4) | 2,179 (20.9) |
| Household |  |  |  |  |  |  |  |  |  |  |  |
| Alone |  | 225 (7.8) | 217 (7.6) | 67 (2.2) | 132 (4.3) | 242 (7.5) | 242 (7.0) | 253 (6.7) | 311 (7.6) | 291 (7.2) | 322 (7.8) |
| Multiple |  | 3,844 (12.7) | 3622 (12.1) | 1023 (3.5) | 1784 (6.3) | 2982 (10.5) | 3013 (10.6) | 3,193 (11.4) | 3,761 (13.7) | 3289 (12.2) | 3,317 (12.5) |
| Residence |  |  |  |  |  |  |  |  |  |  |  |
| Haramachi |  | 2,250 (10.4) | 2172 (10.1) | 671 (3.2) | 1205 (5.8) | 1951 (9.3) | 1986 (9.4) | 2,203 (10.4) | 2,598 (12.1) | 2318 (10.9) | 2,364 (11.2) |
| Kashima |  | 796 (14.6) | 750 (14.0) | 265 (5.1) | 389 (7.5) | 702 (13.6) | 670 (12.8) | 667 (12.6) | 805 (15.2) | 709 (13.5) | 746 (14.4) |
| Odaka |  | 1,023 (17.1) | 917 (15.6) | 154 (2.7) | 322 (5.8) | 571 (10.4) | 599 (11.1) | 576 (11.1) | 669 (13.6) | 553 (12.2) | 529 (12.4) |
| Evacuation status |  |  |  |  |  |  |  |  |  |  |  |
| Inside of the City | Non-evacuee | - | - | 591 (3.90) | 1279 (6.75) | 2177 (11.06) | 2188 (10.82) | 2411 (11.77) | 2815 (13.57) | 2526 (12.17) | 2577 (12.72) |
|  | Evacuee | - | - | 59 (2.81) | 324 (6.51) | 626 (11.27) | 652 (11.20) | 656 (10.72) | 825 (13.00) | 764 (11.31) | 831 (11.50) |
| Outside of the City | Evacuee | - | - | 384 (2.98) | 312 (4.19) | 418 (6.72) | 415 (7.42) | 378 (7.54) | 431 (9.66) | 290 (8.31) | 231 (7.71) |

Number of participants and participation rate (%) of each category are listed.

Supplementary Table3 Multivariable logistic regression analysis for the CRC screening participation, whole period combined

| Variables | Odds (95%CI) | p |
| --- | --- | --- |
| Sex (Reference = Female) |  |  |
| Male | 0.38 (0.35-0.41) | <.001 |
| Age (Reference = 65 – 74yr) |  |  |
| 40 -64yr | 0.33 (0.31-0.34) | <.001 |
| Residence (Reference = Kashima) |  |  |
| Haramachi | 0.59 (0.53-0.65) | <.001 |
| Odaka | 0.98 (0.86-1.12) | .786 |
| Household (Reference = Multiple) |  |  |
| Alone | 0.52 (0.47-0.59) | <.001 |
| Year (Reference = 2018) |  |  |
| 2009 | 0.85 (0.79-0.92) | <.001 |
| 2010 | 0.79 (0.73-0.85) | <.001 |
| 2011 | 0.08 (0.07-0.09) | <.001 |
| 2012 | 0.21 (0.19-0.23) | <.001 |
| 2013 | 0.60 (0.56-0.65) | <.001 |
| 2014 | 0.62 (0.58-0.67) | <.001 |
| 2015 | 0.73 (0.68-0.79) | <.001 |
| 2016 | 1.13 (1.06-1.21) | <.001 |
| 2017 | 0.89 (0.83-0.95) | .001 |

Supplementary Table 4 Multivariable logistic regression analysis for the CRC screening participation after the 2011 Japan triple disaster.

|  | 2011 | | 2012 | | 2013 | | 2014 | | 2015 | | 2016 | | 2017 | | 2018 | | Whole years |  |
| --- | --- | --- | --- | --- | --- | --- | --- | --- | --- | --- | --- | --- | --- | --- | --- | --- | --- | --- |
|  | Odds ^a^ (95%CI) | p | Odds (95%CI) | p | Odds (95%CI) | p | Odds (95%CI) | p | Odds (95%CI) | p | Odds (95%CI) | p | Odds (95%CI) | p | Odds (95%CI) | p |  |  |
| Sex (Reference = Female) | |  |  |  |  |  |  |  |  |  |  |  |  |  |  |  |  |  |
| Male | 0.65 (0.57-0.74) | <.001 | 0.70 (0.63-0.77) | <.001 | 0.64 (0.59-0.69) | <.001 | 0.66 (0.61-0.71) | <.001 | 0.62 (0.57-0.66) | <.001 | 0.54 (0.51-0.58) | <.001 | 0.58 (0.54-0.63) | <.001 | 0.54 (0.50-0.58) | <.001 | 0.34 (0.31-0.37) | <.001 |
| Age (Reference = 65yr – 74yr ^b^) | |  |  |  |  |  |  |  |  |  |  |  |  |  |  |  |  |  |
| 40-64yr | 0.37 (0.32-0.41) | <.001 | 0.30 (0.27-0.33) | <.001 | 0.34 (0.32-0.37) | <.001 | 0.32 (0.30-0.35) | <.001 | 0.33 (0.31-0.35) | <.001 | 0.34 (0.32-0.37) | <.001 | 0.30 (0.27-0.32) | <.001 | 0.30 (0.28-0.32) | <.001 | 0.29 (0.27-0.31) | <.001 |
| Residence (Reference = Kashima) | |  |  |  |  |  |  |  |  |  |  |  |  |  |  |  |  |  |
| Haramachi | 0.62 (0.54-0.72) | <.001 | 0.77 (0.69-0.87) | <.001 | 0.66 (0.60-0.72) | <.001 | 0.72 (0.65-0.79) | <.001 | 0.82 (0.74-0.90) | <.001 | 0.79 (0.72-0.86) | <.001 | 0.79 (0.72-0.87) | <.001 | 0.76 (0.70-0.84) | <.001 | 0.60 (0.54-0.68) | <.001 |
| Odaka | 0.61 (0.49-0.77) | <.001 | 1.02 (0.84-1.23) | .836 | 0.99 (0.85-1.15) | .905 | 1.08 (0.93-1.26) | .293 | 1.18 (1.01-1.37) | .037 | 1.07 (0.93-1.23) | .354 | 1.06 (0.91-1.23) | .429 | 1.02 (0.88-1.18) | .812 | 0.98 (0.83-1.14) | .767 |
| Household (Reference = Multiple) | |  |  |  |  |  |  |  |  |  |  |  |  |  |  |  |  |  |
| Alone | 0.61 (0.47-0.78) | <.001 | 0.63 (0.52-0.76) | <.001 | 0.68 (0.59-0.78) | <.001 | 0.64 (0.55-0.73) | <.001 | 0.58 (0.51-0.67) | <.001 | 0.56 (0.50-0.64) | <.001 | 0.59 (0.52-0.68) | <.001 | 0.63 (0.56-0.72) | <.001 | 0.49 (0.43-0.55) | <.001 |
| Evacuation (Reference = Non-evacuation) | |  |  |  |  |  |  |  |  |  |  |  |  |  |  |  |  |  |
| Evacuee | 0.78 (0.68-0.89) | <.001 | 0.71 (0.62-0.81) | <.001 | 0.70 (0.63-0.78) | <.001 | 0.75 (0.68-0.84) | <.001 | 0.68 (0.61-0.76) | <.001 | 0.78 (0.71-0.86) | <.001 | 0.80 (0.72-0.88) | <.001 | 0.78 (0.71-0.86) | <.001 | 0.65 (0.60-0.71) | <.001 |
| Year (Reference = 2018) | |  |  |  |  |  |  |  |  |  |  |  |  |  |  |  |  |  |
| 2011 | - |  | - |  | - |  | - |  | - |  | - |  | - |  | - |  | 0.08 (0.07-0.08) | <.001 |
| 2012 | - |  | - |  | - |  | - |  | - |  | - |  | - |  | - |  | 0.20 (0.18-0.22) | <.001 |
| 2013 | - |  | - |  | - |  | - |  | - |  | - |  | - |  | - |  | 0.61 (0.56-0.66) | <.001 |
| 2014 | - |  | - |  | - |  | - |  | - |  | - |  | - |  | - |  | 0.62 (0.57-0.67) | <.001 |
| 2015 | - |  | - |  | - |  | - |  | - |  | - |  | - |  | - |  | 0.73 (0.68-0.79) | <.001 |
| 2016 | - |  | - |  | - |  | - |  | - |  | - |  | - |  | - |  | 1.16 (1.08-1.25) | <.001 |
| 2017 | - |  | - |  | - |  | - |  | - |  | - |  | - |  | - |  | 0.88 (0.82-0.95) | .001 |

^a^ Odds: odds ratio, ^b^ yr: years old

Supplementary Table 5 Multivariable analysis with different ways of dividing the type of evacuation

|  | Non-evacuation vs Evacuation | | Non-evacuation vs Inside of the city vs Outside of the city^*^ | | Inside of the city vs Outside of the city^**^ | |
| --- | --- | --- | --- | --- | --- | --- |
|  | Odds ^a^ (95%CI) | P value | Odds (95%CI) | P value | Odds (95%CI) | P value |
| Evacuation status |  |  |  |  |  |  |
| Non-Evacuation | Reference |  | Reference |  | - | - |
| Evacuation | 0.65 (0.60-0.71) | <.001 | - |  | - | - |
| Within the city | - |  | 0.89 (0.81-0.99) | <.001 | - | - |
| Outside the city | - |  | 0.50 (0.46-0.56) | <.001 | 0.52 (0.48-0.57) | <.001 |
| Within the city  (Including non-evacuation) | - |  | - | - | Reference | - |
| Year (Reference = 2018) |  |  |  |  |  |  |
| 2011 | 0.08 (0.07-0.08) | <.001 | 0.09 (0.08-0.10) | <.001 | 0.09 (0.08-0.10) | <.001 |
| 2012 | 0.20 (0.18-0.22) | <.001 | 0.21 (0.19-0.23) | <.001 | 0.21 (0.19-0.23) | <.001 |
| 2013 | 0.61 (0.56-0.66) | <.001 | 0.63 (0.58-0.68) | <.001 | 0.63 (0.58-0.68) | <.001 |
| 2014 | 0.62 (0.57-0.67) | <.001 | 0.64 (0.59-0.69) | <.001 | 0.64 (0.59-0.69) | <.001 |
| 2015 | 0.73 (0.68-0.79) | <.001 | 0.75 (0.70-0.81) | <.001 | 0.75 (0.70-0.81) | <.001 |
| 2016 | 1.16 (1.08-1.25) | <.001 | 1.19 (1.10-1.27) | <.001 | 1.19 (1.10-1.28) | <.001 |
| 2017 | 0.88 (0.82-0.95) | .001 | 0.89 (0.83-0.96) | .002 | 0.89 (0.83-0.96) | .002 |
| Residence (Reference = Kashima) | |  |  |  |  |  |
| Haramachi | 0.6 (0.54-0.68) | <.001 | 0.62 (0.55-0.69) | <.001 | 0.62 (0.55-0.69) | <.001 |
| Odaka | 0.98 (0.83-1.14) | .767 | 0.96 (0.82-1.13) | .629 | 0.91 (0.78-1.05) | .197 |
| Age (Reference = 65 – 74yr ^b^) |  |  |  |  |  |  |
| 40-64 yr | 0.29 (0.27-0.31) | <.001 | 0.28 (0.26-0.3) | <.001 | 0.28 (0.27-0.3) | <.001 |
| Sex (Reference = Female) |  |  |  |  |  |  |
| Male | 0.34 (0.31-0.37) | <.001 | 0.34 (0.31-0.37) | <.001 | 0.34 (0.31-0.37) | <.001 |
| Household (Reference = Multiple) | |  |  |  |  |  |
| Alone | 0.49 (0.43-0.55) | <.001 | 0.49 (0.43-0.56) | <.001 | 0.48 (0.42-0.54) | <.001 |

a Odds: odds ratio, b yr: years old

* Stratified by those not under evacuation, those evacuated within the city, and those evacuated outside the city.

** Comparison of those who evacuated outside the city and those who are in the city including those who did not evacuate and those who evacuated to the city.

Supplementary Table 6 Multivariable logistic regression analysis for the CRC screening participation by pre-disaster screening adherence, whole period combined

|  | participated CRC screening in both 2009 and 2010 | | participated CRC screening either in 2009 or 2010 | | did not participate CRC screening in 2009 or 2010 | |
| --- | --- | --- | --- | --- | --- | --- |
|  | Whole periods | |  |  |  |  |
| Variable | Odds (95%CI) | p | Odds(95%CI) | p | Odds(95%CI) | P |
| Age (Reference = 60-64yr) |  |  |  |  |  |  |
| 40-49 yr | 0.36 (0.25-0.52) | <.001 | 0.17 (0.11-0.27) | <.001 | 0.12 (0.10-0.15) | <.001 |
| 50-59 yr | 0.78 (0.60-1.01) | .061 | 0.60 (0.43-0.83) | .002 | 0.52 (0.44-0.61) | <.001 |
| Sex (Reference = Female) |  |  |  |  |  |  |
| Male | 0.61 (0.47-0.78) | <.001 | 0.69 (0.51-0.94) | .019 | 0.45 (0.39-0.52) | <.001 |
| Residence (Reference = Kashima) |  |  |  |  |  |  |
| Haramachi | 0.82 (0.60-1.10) | .184 | 0.86 (0.59-1.25) | .435 | 0.74 (0.62-0.89) | <.001 |
| Odaka | 0.51 (0.35-0.74) | <.001 | 1.03 (0.63-1.68) | .899 | 0.92 (0.72-1.18) | .512 |
| Household (Reference = Multiple) |  |  |  |  |  |  |
| Alone | 1.05 (0.71-1.55) | .823 | 1.22 (0.76-1.95) | .414 | 0.81 (0.67-0.99) | .037 |
| Evacuation status (Reference = Non-Evacuation) |  |  |  |  |  |  |
| Evacuation | 0.50 (0.40-0.62) | <.001 | 0.50 (0.39-0.66) | <.001 | 0.84 (0.73-0.97) | .014 |
| Year (Reference = 2018) |  |  |  |  |  |  |
| 2011 | 0.09 (0.07-0.12) | <.001 | 0.06 (0.04-0.08) | <.001 | 0.03 (0.02-0.03) | <.001 |
| 2012 | 0.21 (0.17-0.26) | <.001 | 0.12 (0.09-0.15) | <.001 | 0.08 (0.07-0.09) | <.001 |
| 2013 | 0.88 (0.72-1.08) | .210 | 0.57 (0.45-0.71) | <.001 | 0.28 (0.25-0.31) | <.001 |
| 2014 | 0.89 (0.73-1.09) | .271 | 0.59 (0.47-0.73) | <.001 | 0.32 (0.28-0.35) | <.001 |
| 2015 | 0.89 (0.73-1.08) | .242 | 0.74 (0.59-0.92) | .008 | 0.46 (0.42-0.52) | <.001 |
| 2016 | 1.25 (1.02-1.54) | .028 | 1.03 (0.82-1.29) | .794 | 0.91 (0.82-1.00) | .055 |
| 2017 | 0.97 (0.79-1.18) | .753 | 0.70 (0.56-0.88) | .002 | 0.82 (0.74-0.90) | <.001 |

Supplementary Figure1 Transition of evacuation in Minamisoma City


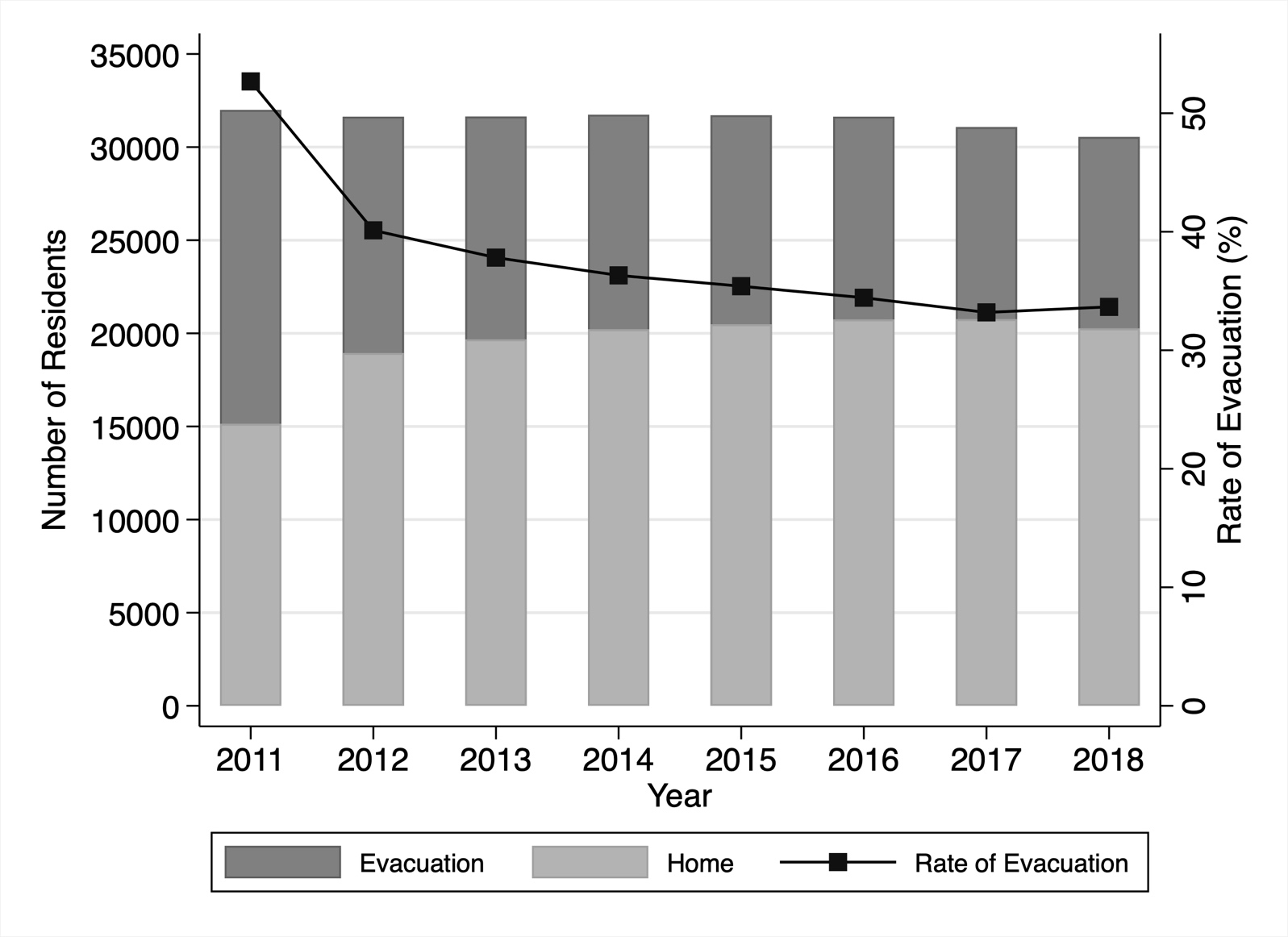


The figure shows the transition between the home and others (evacuation). The darker bars show the number of people who had been evacuated and lived elsewhere, while the lighter bars show the number of people who lived at home. The broken line represents the proportion of the evacuees.

Supplementary Figure2 **Changes in the number of people living with residents**


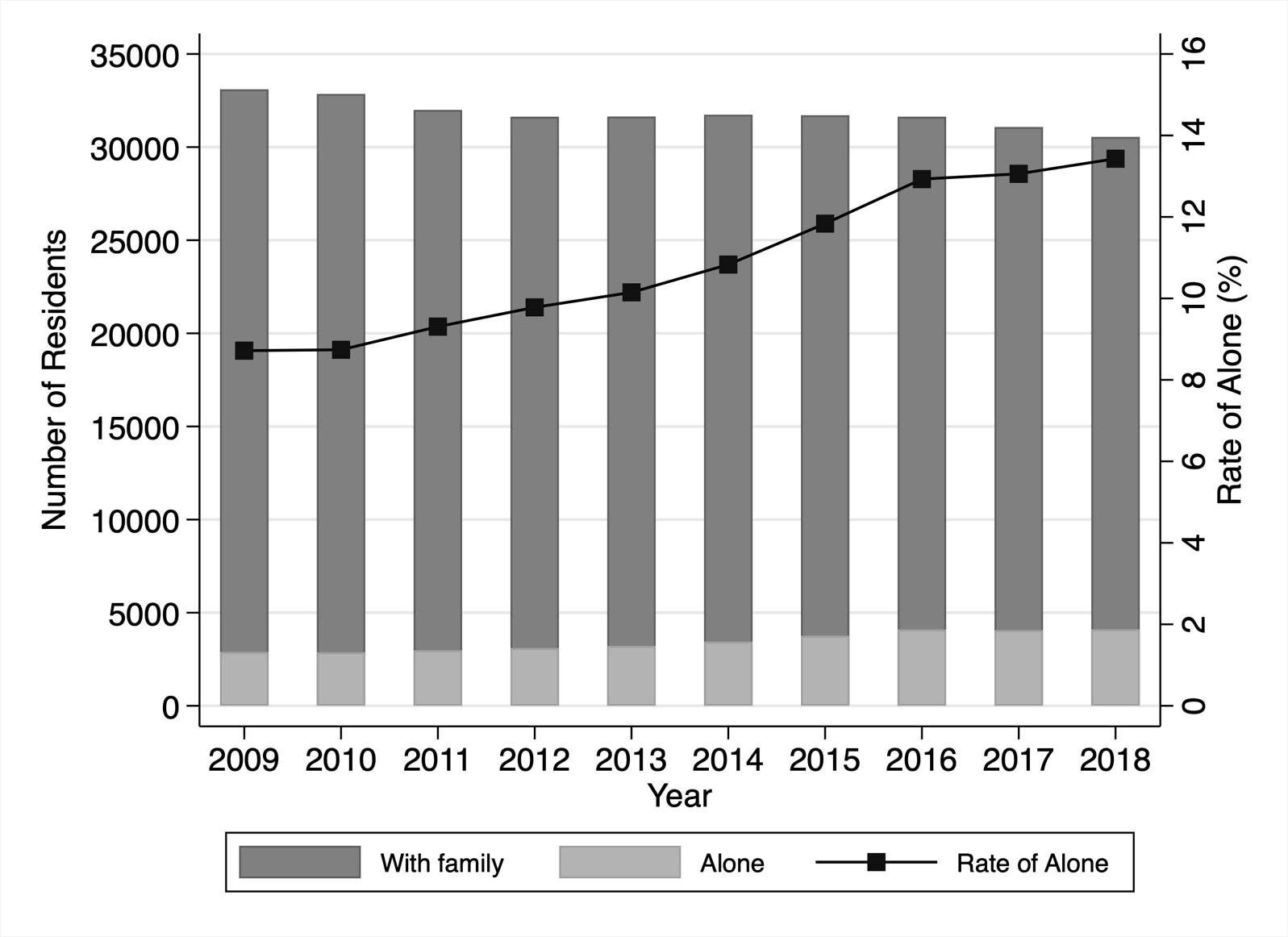


The graph shows the number and percentage of people who live alone and those who share a household with more than one person. Lighter bars indicate the number of single persons, while darker bars indicate the number of people in households with more than one person, such as families. The line graph shows the proportions of single persons.
